# Enhanced quantification for 3D SEM–EDS: Using the full set of available X-ray lines

**DOI:** 10.1016/j.ultramic.2014.10.010

**Published:** 2015-01

**Authors:** Pierre Burdet, S.A. Croxall, P.A. Midgley

**Affiliations:** Department of Materials Science and Metallurgy, University of Cambridge, Charles Babbage Road 27, Cambridge, CB3 0FS Cambridgeshire, UK

**Keywords:** Energy dispersive X-ray spectrometry, Focused ion beam, Tomographic spectral imaging, 3D chemical analysis, 3D microanalysis, Quantification, 3D image analysis

## Abstract

An enhanced method to quantify energy dispersive spectra recorded in 3D with a scanning electron microscope (3D SEM–EDS) has been previously demonstrated. This paper presents an extension of this method using all the available X-ray lines generated by the beam. The extended method benefits from using high energy lines, that are more accurately quantified, and from using soft X-rays that are highly absorbed and thus more surface sensitive. The data used to assess the method are acquired with a dual beam FIB/SEM investigating a multi-element Ni-based superalloy. A high accelerating voltage, needed to excite the highest energy X-ray line, results in two available X-ray lines for several elements. The method shows an improved compositional quantification as well as an improved spatial resolution.

## Highlights

• 3D SEM–EDS mapping of structures smaller than the X-ray generation volume is considered.
• The influence of neighbouring voxels is corrected by a recursive quantification method.
• The method is improved using all available X-ray lines.
• The method is applied to simulated spectra and experimental dual beam FIB/SEM data.
• The method is shown to improve both compositional accuracy and spatial resolution.

## 1 Introduction

For modern scanning electron microscopes (SEMs) working at low accelerating voltages (below 1 kV), the interaction volume may be sufficiently small for secondary electrons, back-scattered electrons or a mixed signal that the spot size may then be the limiting factor for image resolution. However, when elemental analysis is needed, using for example an energy dispersive X-ray spectrometer (EDS), a higher accelerating voltage is required to excite all the X-ray lines of interest. With an accelerating voltage greater than 10 kV the interaction volume is, in general, dramatically larger and typically in the micron range. The large interaction volume is therefore the main limitation for SEM–EDS in terms of the size of features that can be analysed accurately.

With an EDS detector, X-rays can be recorded over a wide range of energies, allowing almost all elements to have at least one characteristic X-ray line in an EDS spectrum. This makes EDS a versatile tool for a wide range of materials, especially those composed of many elements. With such samples, a high accelerating voltage is needed to ensure that all X-ray lines of interest are detected and the constituent elements are likely to generate X-rays from different inter-shell transitions: the Kα, Lα, Mα are of greatest use.

**Fig. 1:**
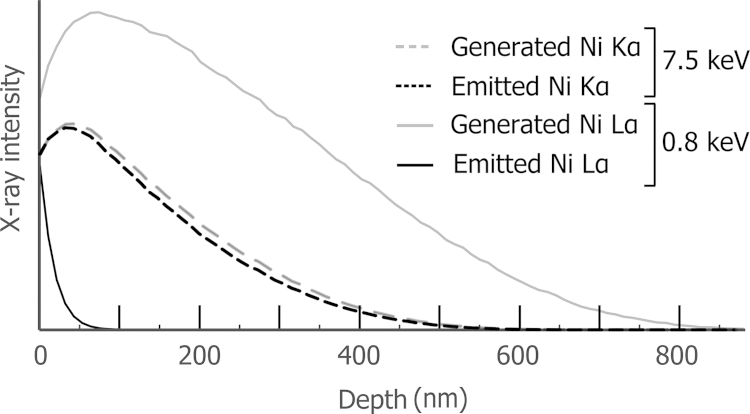
Simulated X-ray depth distributions of Ni Kα and Ni Lα at 15 kV. The simulation parameters correspond to a typical 3D EDS acquisition. The sample has the composition of the γ′ phase of a Ni-based superalloy which is approximately Ni_3_(Al,Ta,Ti). The surface normal is tilted 38° with respect to the beam.

To illustrate the difference between two main lines, a specific example is considered in Fig. 1 for Ni Kα and Ni Lα for a Ni-base superalloy (RR1000) [1]. In this figure, a simulated X-ray intensity is plotted as a function of the depth in the sample. The probability of generating X-rays is directly linked to the probability of inner shell ionisation (see [2] for more details). As the electron beam travels deeper into the sample it continuously loses energy and intensity, and is less likely to ionise an atom. To be ionised the L shell needs less energy than the corresponding K shell and Lα X-rays are generated from deeper in the sample, as observed in Fig. 1.

The emitted intensity of an X-ray depends on the fraction of X-rays that travel though the sample towards the detector without absorption. Following Beer׳s law [2], the emitted intensity is linked to the incident intensity by the exponential of the mass absorption coefficient. Since the energy difference among the Kα, Lα and Mα X-ray lines is several keV, the mass absorption coefficient for the lower energy line is likely to be several orders of magnitude higher [3]. The difference in absorption between two main lines can be extreme as observed in Fig. 1. Ni Lα X-rays are emitted from only the first 100 nm below the surface and thus absorption acts as a depth filter. A Ni Lα element map then has a significantly improved depth resolution compared to the corresponding Ni Kα map.

Low energy lines therefore seem to be the lines to choose for EDS analysis in terms of spatial resolution. However, some drawbacks have to be considered. With the limited energy resolution of an EDS detector, the low energy lines are more likely to overlap in the spectrum. A deconvolution method is needed that acts as a source of uncertainty. The continuous X-ray background in the low energy range is more complex and that results in a higher uncertainty when correcting for it. When quantifying an EDS spectrum intensity, absorption needs to be corrected and this can be the most signification correction [2]. The higher the accelerating voltage, the longer the X-ray path to the surface and the larger the absorption correction and the resulting uncertainty. The mass absorption coefficient database for soft X-rays is of lower quality, as they are more complex to measure accurately [4], which results in higher uncertainty for absorption correction [5]. With improved spatial resolution, but a lower compositional accuracy, the low energy X-ray lines can therefore be regarded as complementary to the high energy lines.

Classical quantification procedures, such as the well-established XPP-*φ*(*ρz*) method [6], use one line per element and assume a homogeneous sample over the full range of X-ray generation [2]. They cannot be used when the sample microstructure is smaller than the interaction volume, which is when the improved spatial resolution of low energy X-ray lines becomes valuable. Some methods have been extended to include heterogeneous samples of known microstructure, such as fibres and particles on a substrate [7], [8], spheres embedded in a matrix [9] and thin film layers on a substrate [10], [11], [12]. In [10], the approach to extend the XPP-*φ*(*ρz*) method to thin film quantification is to iteratively predict and refine a model of the sample. Such a method can take advantage of all the available lines for a more reliable convergence [13].

For a quantification method adapted to a sample of unknown microstructure, that microstructure has to be estimated from the EDS mapping itself. The mapping needs to contain information in all three dimensions and especially in the depth direction, the primary direction of the incident high energy electrons and thus the direction of the broadest X-ray distribution. Using 3D SEM–EDS data, a quantification method was developed for heterogeneous samples of unknown microstructure [14]. Called enhanced quantification, this method corrects the influence of the neighbouring voxel, applying recursively the XPP-*φ*(*ρz*) thin film quantification [10]. This method is the focus of the present paper.

**Fig. 2:**
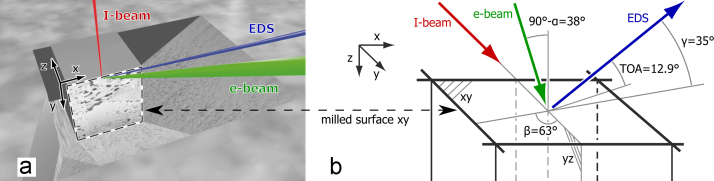
Geometry for 3D SEM–EDS acquisition. (a) shows a schematic view of a sample. The sample surface is tilted to be perpendicular to the ion beam. The freshly milled surface that is analysed is surrounded by a dashed box. A protective layer is deposited on the block of interest. The *z* axis shows the direction of sequential milling. (b) gives details of the geometry. The two beams are in the *yz*-plane with an angle *α* of 52° between them. The ion beam is parallel to the milled surface (*xy*-plane). The electron beam is tilted 38° with respect to the normal of the *xy*-plane. *β* is the azimuth angle, the angle between the EDS detector and the ion beam direction projected on a surface tilted 0°. *γ* is the elevation angle of the EDS detector, the take-off angle for a surface titled 0°. The take-off angle (TOA) for the milled surface (tilted 38°) is given.

SEM–EDS mapping can be extended to three-dimensional (3D) microanalysis using a dual beam microscope formed by a SEM coupled to a focused ion beam (FIB) [15], [16], [17]. The geometry of acquisition is shown in Fig. 2. In a “slice-and-view” approach, a thin layer of material is milled away by the ion beam, and the freshly milled surface (dashed line in Fig. 2(a)) is characterised by SEM imaging and 2D EDS mapping. The recorded 3D data is composed of a stack of SEM images and a stack of EDS maps, so-called “spectrum-images”.

Compared to 2D EDS mapping, 3D EDS mapping suffers from specific limitations due to time constraints and to the complex acquisition geometry seen in Fig. 2(a). With long acquisition times the overall conditions might change, inducing a specimen drift that can affect the milling and image acquisition. To cover a large volume in a reasonable time the dwell time per spectrum is reduced, leading to individual spectra with a low total of X-rays counts. In an acquisition without stage movement, the analysed surface is inclined and the take-off-angle is low. The analysed surface is surrounded typically by trenches, in which spurious X-rays may be emitted. Minimising these limitations during acquisition and through post-processing, 3D EDS by FIB/SEM was shown to be a powerful tool to enable a deep understanding of a sample [15], [18].

The present work introduces a new version of the enhanced quantification for 3D SEM–EDS data proposed in [14]. This method is improved to take advantage of all available X-ray lines, benefiting from the higher quantification accuracy of the high energy X-ray lines and the improved spatial resolution of the low energy X-ray lines. To assess the method, a Ni-based superalloy sample is used containing 11 elements. In a first step, the method is applied to 3D EDS data simulated in a geometrically simple case. It is then applied to 3D EDS data acquired on a FIB/SEM. The method is assessed in comparison with the classical method and with references sample, demonstrating the benefit of using the full set of available lines.

## 2 Materials and methods

### 2.1 Ni-based superalloy

The sample used for the present investigation is a Ni-based superalloy (RR1000). The alloy was produced via powder metallurgy and was given a super-solvus heat treatment. Forged RR1000 was solution heat-treated at 1170 °C for 4 h, cooled at 1 °C/min, and then aged for 12 h at 800 °C. The cooling rate, notably slower than those in disc forgings, was chosen to yield large precipitates that show dendritic morphology at a scale suitable for the present investigation.

**Fig. 3:**
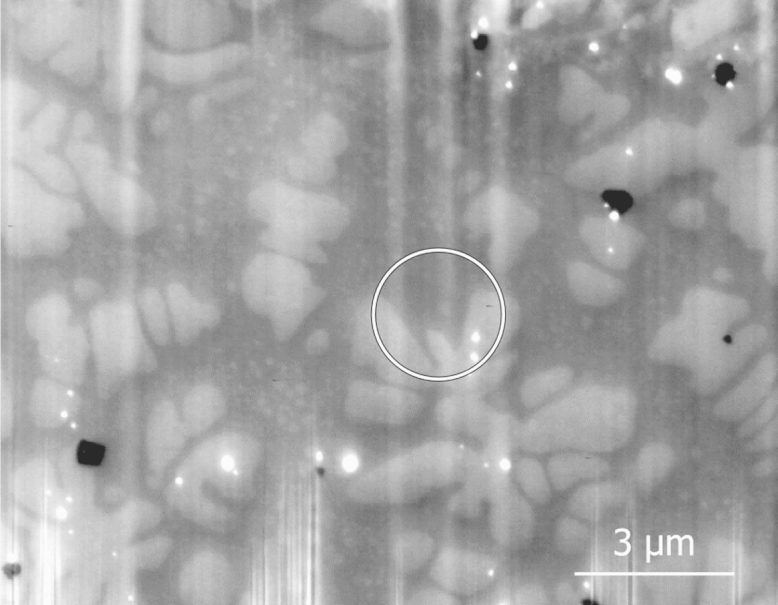
Secondary electron (SE) micrograph of a Ni-based superalloy sample. The image, one of a series used for 3D reconstruction, is recorded at 15 kV with a through-the-lens SE detector. The surface is polished by FIB milling. The vertical lines are polishing artefacts, due to the “curtaining effect”. The white circle indicates a γ′ dendrite arm buried under the surface, see also Fig. 9.

The alloy is composed of 11 different elements: Ni, Co, Cr, Mo, Ti, Al, Ta, Hf, Zr, O and C. A secondary electron (SE) image of the 3D acquisition is shown in Fig. 3. Apart from the vertical lines due to a FIB polishing artefact the contrast seen in this image is linked to compositional variations, as confirmed by EDS. The sample is formed of two main phases, a γ-matrix in dark grey and a second γ′ phase in light grey, and two minority phases seen as small precipitates with bright and dark contrast. The γ-matrix is rich in Ni, Co, Cr and Mo and the γ′ phase is Ni_3_(Al,Ta,Ti). The black precipitates are carbides rich in Ti, Ta and Hf. The white precipitates are rich in Hf and likely to be an oxide. A more detailed description of the different phases can be found in [1].

### 2.2 3D EDS microanalysis

The FIB/SEM dual beam used in this study is a FEI Helios Nanolab. The angle between the gallium ion beam and the electron beam is 52°. The microscope is fitted with an X-max 80 mm^2^ EDS detector from Oxford Instruments. The geometry of acquisition is shown in Fig. 2(a). The sample is tilted to obtain a surface perpendicular to the ion beam. Trenches are milled away at the side of the volume of interest to avoid redeposition. The freshly milled surface surrounded by a dashed line in Fig. 2(a) is the analysed surface. With the milled surface perpendicular to the sample surface the milled surface is tilted 38° with respect to the beam and the X-rays leave this surface towards the EDS detector with a take-off angle of 12.9°, as shown in Fig. 2(b).

The optimised EDS acquisition parameters are given in Table 1. The accelerating voltage (*V*_0_) of 15 kV is the lowest with which the full set of X-ray lines is efficiently excited. To increase the count rate the detector process time is lowered, still keeping a sufficient energy resolution. The current is set in order to obtain a detector saturation of 50%. The time to record one spectrum, the dwell time, is reduced to obtain several imaging/milling sequences per hour. The slice thickness of 100 nm is chosen based on the simulation in Fig. 1, and the pixel size is set to half that value. During the EDS map acquisition, drift is compensated every 2 min by cross-correlation of the SE images. The SE images, see Fig. 3, were acquired using the detector inside the electron column (through the lens detector) and immersing the sample in a magnetic field originating from the electron column. The field is switched off during milling. The pixel size of the SE image is set 4× smaller than the EDS maps. Standard spectra used for quantification were recorded with the same microscope parameters, apart from a longer dwell time of 30 s. The surface of the standard is tilted 38° and pure materials mounted on a separate support are used.

**Table 1:** Acquisition parameters of the 3D SEM–EDS acquisition. V0 is the accelerating voltage. “Dwell time” is the acquisition time for a single spectrum. “Rate“ is the count rate (rate of detected X-rays). “Slice *t*.” is the slice thickness.

*V*_0_	Rate	Dwell time	Slice *t*	Pixel size
15 kV	38 kct/s	18 ms	100 nm	50×50 nm^2^

**Fig. 4:**
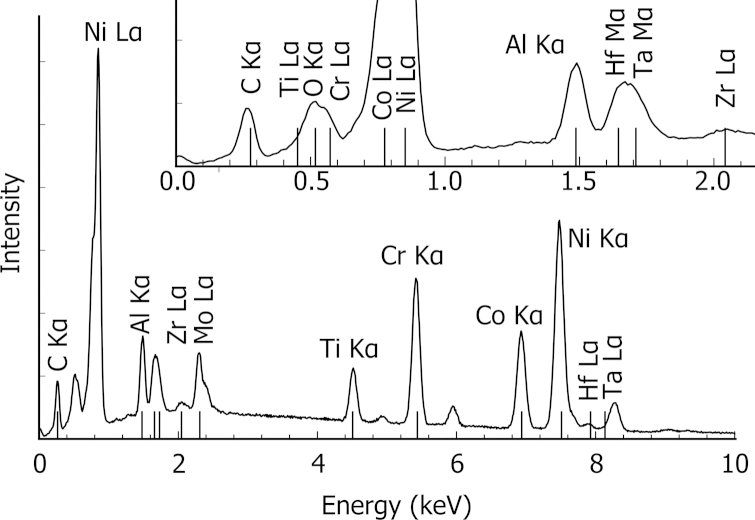
Characteristic EDS spectrum acquired from the Ni-based superalloy sample. The main X-ray lines excited at 15 kV are indicated. The EDS spectrum is a sum over spectra recorded using the parameters detailed in Table 1. The inset shows a magnification of the low energy lines. The full set of lines used for quantification are shown. Minor lines are not labelled.

In Fig. 4, the sum of 1000 spectra is shown. X-ray lines for all 11 elements of the Ni-based superalloy can be observed. Five elements (C, O, Al, Zr, Mo) have one resolved main line (α) between 0 and 15 keV, and the other elements have two major lines. Severe overlaps are observed for Hf Mα/Ta Mα, Co Mα/Ni Mα, and Ti Lα/Cr Lα/O Kα. Ti Lα is in the low energy region where the detector has a relatively low sensitivity. This peak severely overlaps with other minor peaks not shown in Fig. 4 and is not used for quantification. Oxygen is present only in the small phase of Hf oxide and oxygen content is obtained assuming stoichiometry with Hf. Two main lines are used for five elements, giving a total of 15 X-ray lines used in the analysis.

The data were acquired using the FEI software package for 3D SEM–EDS, namely EDS3. The intensities were extracted by the method described by Goldstein et al. [2]. The background is reduced by applying a top hat filter. Spectra of known composition measured on the standards are fitted by least squares to spectra of the superalloy sample. The quantification (enhanced and bulk) was applied using the library of Stratagem^®^, a commercial software based on the work of Pouchou and Pichoir [6], [10]. The Ni elemental maps from each slice were registered using a cross-correlation approach developed by Thèvenaz et al. [19] and were used as a reference to align the whole data set. Principal component analysis (PCA) was applied to the data to reduce the noise using hyperspy, a python-based software for hyperspectral data processing [20]. Monte Carlo simulations were run with the NIST-Monte library by Ritchie [21]. Ten thousand electron trajectories were used per spectrum. Hyperspy and python were used as a central processing platform to control the different libraries.

## 3 Calculation

Described in [14], the enhanced quantification for 3D EDS–SEM is revisited and augmented here. The standard quantification, called here bulk quantification, is first considered. For each element of the unknown spectrum, a X-ray line is chosen and compared to the same X-ray line measured on a standard of known composition. The obtained ratio is called the *k*-ratio:

(1) $$k{-\text{ratio}}_{A}=\frac{I_{unk,A}}{I_{std,A}},$$

where *I* is the intensity, *A* is an element, *unk* is the unknown, and *std* is the standard. In a first step to obtain the *k*-ratio, the background is subtracted applying a top hat filter on both spectra. The standard spectrum is then fitted by least squares to the unknown spectrum [22]. Overlapping peaks are deconvolved by the fitting.

The *k*-ratio differs from the actual composition as the unknown and the standard have different compositions. This causes the so-called matrix effects that result from variation in X-ray ionisation (*Z*), in absorption (*A*) and in fluorescence (*F*). The composition is obtained applying correction factors to compensate for each of these effects. More generally,

(2) $$C_{A}=ZAF_{A}k{-\text{ratio}}_{A}\to C=f_{bulk}(k-\text{ratios}),$$

where *C* is the composition, *Z*, *A* and *F* are the correction factors and *f*_*bulk*_ is the correction method. In this work, the well-regarded XPP-*φ*(*ρz*) correction method from Pouchou and Pichoir [6] is used. This method, as with others, assumes a locally homogeneous sample.

The XPP-*φ*(*ρz*) method has been extended to heterogeneous samples for the case of thin films deposited on a substrate [10]. In the general approach, a system of layers (thickness and composition) is established with known parameters and with guesses for unknown parameters. Varying the unknowns, the system is refined through an iterative procedure until a convergence criterion is reached. In the unfavourable cases of layers sharing common elements, the iteration may converge to a local minimum or not converge in the limit of the iteration step. To avoid such cases, measurements from several accelerating voltages should be used.

**Fig. 5:**
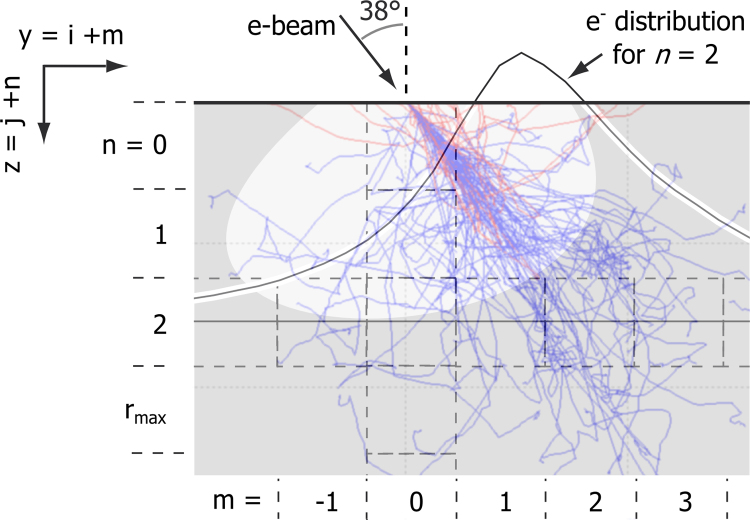
Schematic 3D EDS acquisition. The electron beam is tilted 38° with respect to the surface normal. A system of voxels is drawn with dashed boxes. An indexing system for y and z axes is defined. The central voxel is indicated by indices *i,j* and *m,n* are indices relative to this voxel. The trajectories of backscattered electrons and of absorbed electrons are plotted. The electron distribution in the layer *n*=2 is plotted as a function of *m*.

The XPP-*φ*(*ρz*)) method has been further extended to samples of unknown microstructure for 3D SEM–EDS data. The effect of the surrounding voxels is taken into account extending Eq. (2) as follows:

(3) $$C_{i,j}=f_{layers}(k{-\text{ratios}}_{i,j},{\bar{C}}_{j+1},{\bar{C}}_{j+2},\ldots ,{\bar{C}}_{j+n},\ldots ,{\bar{C}}_{j+r_{max}})$$

where *f*_*layers*_ is the correction method for a layered system, the XPP-*φ*(*ρz*) method for thin film in the present case. The indices *i*,*j* indicate the position of the considered voxel and *m*,*n* indicate the voxel position relative to *i*,*j*, as defined in Fig. 5. *r*_*max*_ is defined as the deepest layer in which the X-rays are generated.$\;{\bar{C}}_{j+n}$ is the local composition seen by the electrons in layer *n*. It is approximated by a weighted average over the neighbouring voxels in this layer. The weighted law is derived from simulated electron distributions, such as the one shown in Fig. 5. Eq. (3) is a recursive relation that can be applied on the data and the global recursion forms the correction method, called enhanced quantification. The recursion is improved by identifying similarities between neighbouring voxels in the depth direction. Layers of similar composition are grouped together in order to simplify the layer system.

Pouchou et al. demonstrated that using the full set of available lines helps avoid local minima in quantifying thin films with the XPP-*φ*(*ρz*) method [13]. The full set of available lines can be used to further improve the enhanced quantification. Eq. (3) is modified to integrate several *k*-ratios per elements.

## 4 Results

### 4.1 Simulations

**Fig. 6:**
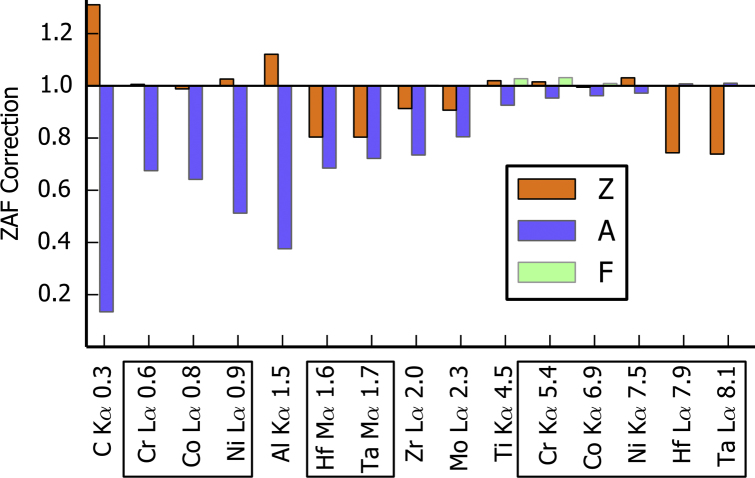
Details of the bulk quantification of one EDS spectrum. The spectrum is simulated at 15 kV in a sample with the composition of the γ-matrix. The ZAF correction factors are plotted for each element (see Eq. (2)). *Z* is the mean atomic number correction (the X-ray spatial distribution correction), *A* is the absorption correction and *F* is the fluorescence correction. The elements with two main X-ray lines are boxed. The energy in keV is given for each X-ray line.

The bulk quantification of one simulated spectrum is first considered in Fig. 6. The quantification is applied to the set of low and high energy lines. The five elements with two main lines are shown boxed. The *ZAF* correction factors applied to the *k*-ratios to obtain the element content, see Eq. (2), is given in Fig. 6. The most important correction factor is the absorption (*A*). For this example, the mean atomic number correction (*Z*) is smaller but still important. The fluorescence correction (*F*) is low in this system and considered negligible for this study. In general, *A* is related to the energy of the lines, a higher correction being required for a lower energy. With pure standards, *Z* is related to the density of the pure element. The density of the superalloy sample is approximately the density of pure Ni. *Z* is greater than one for a lower standard density and less than one for a higher standard density. The X-ray lines with high energy and with a standard density close to the sample density, such as Ni Kα, need almost no correction. The *k*-ratios obtained with such lines are close to the element content and the accuracy of the quantification process is high.

**Fig. 7:**
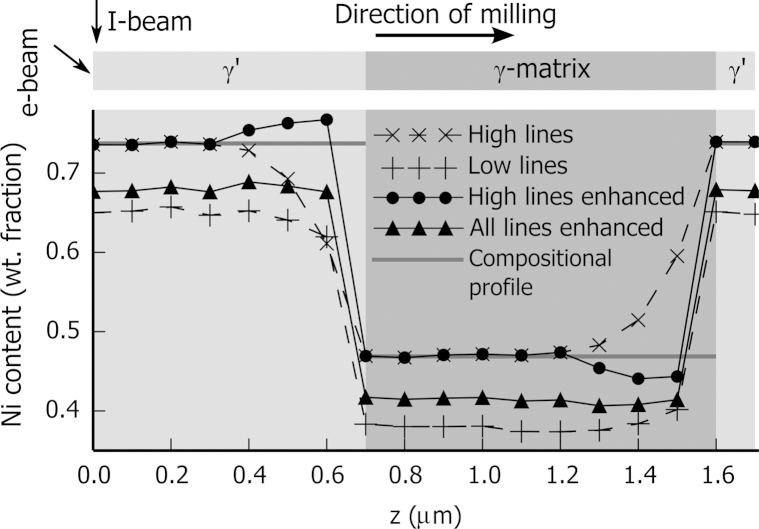
Enhanced quantification applied to a simulated profile along *z*. The geometry is defined in the upper part of the figure. The sample is composed of two layers (γ′ phase on a layer of γ-matrix) on the left sitting on a substrate on the right (γ′ phase). The position of the electron beam and the ion beam is indicated in the top left corner. Every 100 nm along the milling direction, an EDS spectrum is simulated for beam voltage of 15 kV. In the lower part of the image, the profile of the Ni content is plotted for the bulk quantification applied to the set of low and high energy lines (dashed lines) and for the enhanced quantification applied to the set of high and all energy lines (solid lines). The Ni content of the phases is indicated by the horizontal grey lines.

As a first step in the process of enhanced quantification, a simplified model of the 3D acquisition is simulated and shown in Fig. 7. The acquisition is reduced to the depth axis, forming a profile along *z* as described in the upper part of Fig. 7. In the figure the surface of the sample is vertical at *z*=0 nm, and the depth of the sample lies horizontally increasing along *z*. The sample is formed of a layer of γ′ phase on a layer of γ-matrix, itself on a substrate of γ′ phase. Indicated at the top left, the electron beam (e-beam) reaches the vertical surface at an angle of 38°. The ion beam (I-beam) is parallel to the surface. The direction of milling is in the depth direction as indicated. A spectrum is simulated every 100 nm along the milling direction. In the lower part of Fig. 7, the Ni content is given as a function of the position in *z*. Bulk quantification for the set of high and low energy lines (dashed line) is compared with enhanced quantification for the set of high and all energy lines (solid line).

For both types of quantification with the set of high energy lines the Ni content far from the boundaries is the closest to the correct composition, indicated by the horizontal grey lines. The enhanced quantification with the set of all energy lines shows improved Ni content compared to the bulk quantification with the set of low lines. Considering the location nearer the boundary on the smaller *z* side, the influence of the deeper layer is observed resulting in a bent compositional curve for the bulk quantification. The range of influence is greater for the high energy lines than for the low ones. The enhanced quantification helps correct this bending resulting in a squarer profile. In the case of the set of high energy lines the bending is over-corrected, but with the set of all energy lines a square profile is observed.

### 4.2 Measurements

**Fig. 8:**
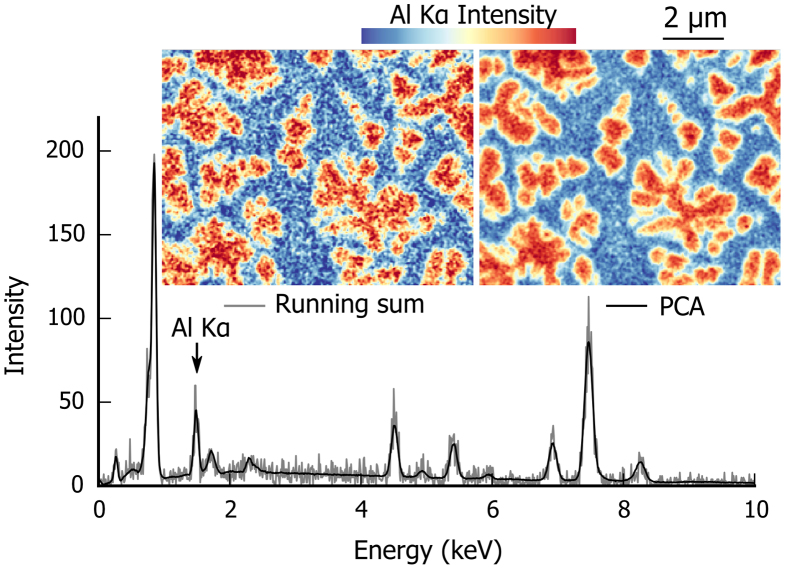
The two processing steps of noise reduction: (i) A running sum with a kernel formed by eight neighbouring pixels in *x* and *y* and (ii) PCA decomposition/reconstruction. A spectrum is plotted before (grey) and after PCA decomposition (black). The spectrum is extracted from the data set at the position of the γ′ phase. Scaled by a colour code, the intensity map for Al Kα is plotted before and after PCA decomposition. (For interpretation of the references to colour in this figure legend, the reader is referred to the web version of this article.)

The time spent per spectrum is set to a short value in order to cover a large volume in a reasonable time. The raw spectra are therefore noisy with a mean number of counts per channel less than one. Before any quantification, effective smoothing is needed; Fig. 8 illustrates the smoothing method. First, a running sum is applied to the data with a square kernel formed by the eight neighbouring pixels. The set of data contains millions of spectra characterising only a limited set of chemical phases. This is a favourable case for a multivariate statistical approach such as principal component analysis (PCA). Using PCA, the set of spectra is decomposed and then a model of the data is reconstructed leaving out the components characteristic of the noise [23]. The singular value decomposition (SVD) method was used to perform the PCA decomposition. Prior to the decomposition, the data were scaled to take into account Poisson statistics [24]. By inspection of the scree plot and the noise content in the individual components, the first nine components were chosen to reconstruct the model. As seen in Fig. 8, the PCA-adjusted spectrum is relatively noise-free. In the intensity map, features appear more clearly and can be correlated to the SE images of Fig. 3. PCA is not free of artefact however and the reconstruction has to be inspected carefully for each new set of data. An artefact is observed if PCA is applied on the raw data before the running sum: as the signal-to-noise ratio is not sufficiently high in the peak formed by the strongly overlapping Hf Mα and Ta Mα lines, the precise peak shape needed for deconvolution is lost during PCA reconstruction and the Hf and Ta maps look identical.

**Fig. 9:**
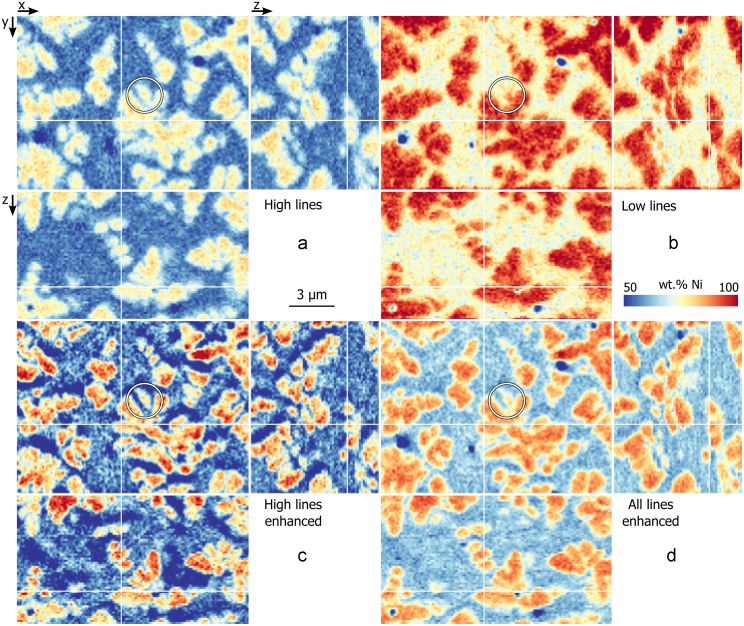
Comparison between quantification methods. The coloured maps give the Ni content as orthogonal sections of the 3D stack. The *xy* image is the same as the SE image of Fig. 3. The Ni content was obtained with the bulk quantification in the upper row and with the enhanced quantification for the lower row. Map a and map c are quantified with the set of low lines, map b with the set of low lines, and map d with the all energy lines. The white circle indicates a γ′ dendrite arm buried under the surface. The white lines show the position of the orthogonal sections. (For interpretation of the references to color in this figure legend, the reader is referred to the web version of this article.)

After noise filtering, the *k*-ratios are extracted and the *k*-ratio maps are aligned by cross-correlation. The different quantification methods are then applied. Fig. 9 shows the quantified Ni content for one map of the 3D stack. The upper row is quantified with the bulk method for the set of high and low energy X-ray lines, respectively (maps a and b).

The bulk quantification with high energy lines (map a) shows a lower Ni content than the bulk quantification with low lines (map b). This is the opposite of that observed in the simulation of Fig. 7: the simulated *k*-ratios of Ni Lα are smaller than the experimental ones by about 25%. This is explained by the difference in the acquisition geometry between the sample and the standard. The analysed sample surface is surrounded by trenches as shown in Fig. 2. The spurious X-rays emitted from these trenches are added to the measured spectra, increasing the *k*-ratios. A second reason is that the analysed surface is not milled perfectly perpendicular to the sample surface. An angle of 92.5° was measured and thus the analysed surface had a tilt of 35.5° (instead of 38°) and a take-off angle of 14.5° (instead of 13.9°). Even with this small difference in take-off angle, the resulting decrease in absorption is relatively high. With these new angles, the simulated intensity of the Ni Lα is 14% higher and the Ni Kα is 1% higher.

In Fig. 9, the lower row is quantified with the enhanced method with the set of high energy lines and all energy lines (maps c and d, respectively). The contrast of these maps is closer to the bulk-quantified one using high energy lines (map a), whose *k*-ratios are more accurately measured, as just shown, has a lower correction (see Fig. 6), and is thus closer to the true composition. Map c shows a lower Ni content in the matrix regions close to the dendrites. The enhanced quantification tends to over-correct in the direction of smaller *z* and smaller *y*, the direction opposite to the electron beam. The same artefact is observed with the simulation of Fig. 7. This artefact is not observed when using all X-ray lines.

In both enhanced quantified maps (maps c and d), boundaries between the γ matrix, the γ′ dendrites and the carbides (with low Ni content) are better defined than in map a. The white circle shows a dendrite arm that is buried under the surface and is not present in the SE image of Fig. 3 (SE are emitted from close to the surface). This arm is present in the map using high energy lines (map a) but not in the map using low energy lines (map b) as the high energy Ni Kα are emitted from a deeper level. For both enhanced quantified maps (maps c and d) the buried arm appears smaller than in map a, indicating an improved spatial resolution.

**Fig. 10:**
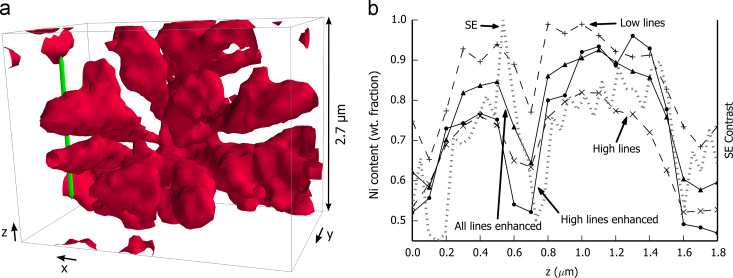
Details of the 3D measurements. (a) The surface, γ' arms, is reconstructed from the stack of Ni content maps obtained with the enhanced quantification applied to all available lines. The line indicates the position of a profile along z shown in (b). This profile goes through two arms of the γ' phase (higher Ni content). The arms are extended in x and y direction and thin in the z direction. The different phase boundaries are approximately perpendicular to z. The SE contrast in dashed grey is adjusted between 0 and 1 (right scale). The Ni content obtained with bulk and enhanced quantification are indicated with dashed and plain curves respectively.

Fig. 10(a) shows details of a 3D reconstruction using the stack of Ni content maps obtained with the bulk quantification applied to the set of high energy lines. The red isosurface indicates a high Ni content (above 70 wt%) and shows part of the γ′ dendrite. The surrounding γ-matrix is transparent. At the left centre of the figure, two dendrite arms of γ′ are seen with ca. 300 nm of γ-matrix in between and the green line indicates the position of a profile parallel to *z* going through the two arms. The different interfaces γ′/γ of the profile are approximately perpendicular to the *z* direction; the profile is analogous to the simulation of Fig. 7. The Ni content of the profile is plotted in Fig. 10(b).

In Fig. 10(b), the SE signal scaled from 0 to 1 is given in dashed grey. The actual position of the phase is not known, but as the SE images have a significantly better resolution, the contrast variation of the SE profile gives a good approximation of the limit of the γ′ phase. Compared to the bulk quantification with the set of high energy lines, the bulk quantification with the set of low energy lines shows a sharper edge at the phase boundaries and a stronger correlation with the SE curve. As discussed before, the composition profile using the set of low energy lines shows a higher Ni content than the profile using the set of high energy lines. The enhanced quantification with all lines lies in-between the two bulk quantified curves and shows similar edge sharpness to the quantification with the set of low energy lines. The enhanced quantification with the set of high energy lines shows higher variation especially on the left of the phase boundaries, corresponding to over-correction. The previous observations can be linked to those observed in the simulation of Fig. 7. A reduction in the effect of noise is also evident: the enhanced quantification with the set of all lines shows the smoothest profile.

**Fig. 11:**
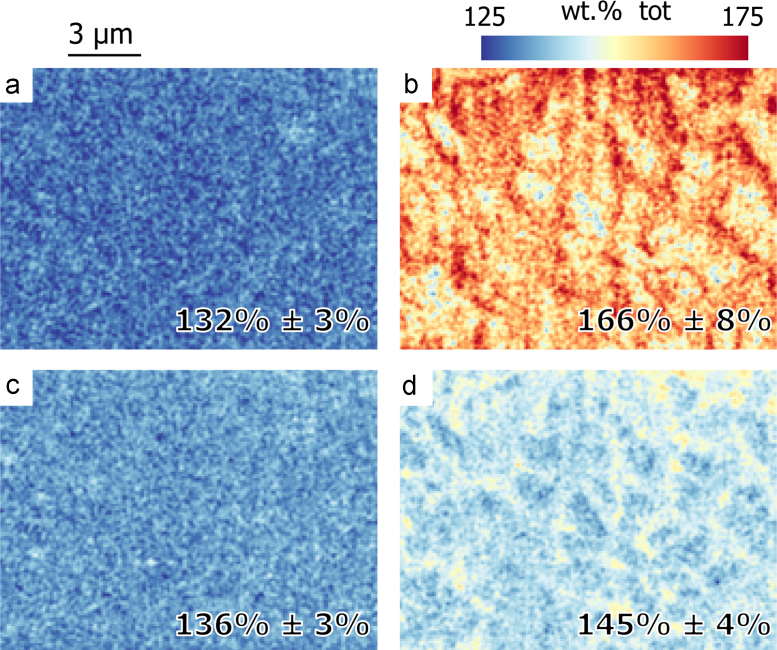
Maps of the analytical total. For each voxel the element contents are summed and the obtained map is plotted with a coloured scale. The same view and same quantification methods as the image *xy* of Fig. 9 is shown. The analytical total was obtained with the bulk quantification for map a and map b and with the enhanced quantification for map c and map d. Map a and map c are quantified with the set of low lines, map b with the set of low lines, and map d with the all energy lines. The mean and the standard deviation are given for each image. (For interpretation of the references to colour in this figure legend, the reader is referred to the web version of this article.)

For the same plan view as in Fig. 9 Fig. 11 gives, for each pixel, the sum over all elements, the so-called “analytical total” [2]. A total of 100% is an indication of an accurate quantification. The same quantification as in Fig. 9 is shown: bulk quantification for maps a (high energy lines) and b (low energy lines), enhanced quantification for maps c (high energy lines) and d (all lines). A total higher than 100% is observed for all maps. As explained earlier, this is due to high *k*-ratios resulting from a difference in the acquisition geometry between the standard and the sample and low energy lines are more affected than high energy lines. With an analytical total closer to 100%, a low standard deviation and little contrast across the map, an improved quantification is obtained with the set of high energy lines (map a and c). Using the set of all lines gives a map d with higher value and with higher contrast. With the set of low energy lines, map b shows the highest contrast and the highest total. The contrast can be linked to the interface between γ′ dendrites and γ-matrix where absorption conditions are different. The same contrast, albeit much weaker, can be observed in map d.

**Fig. 12:**
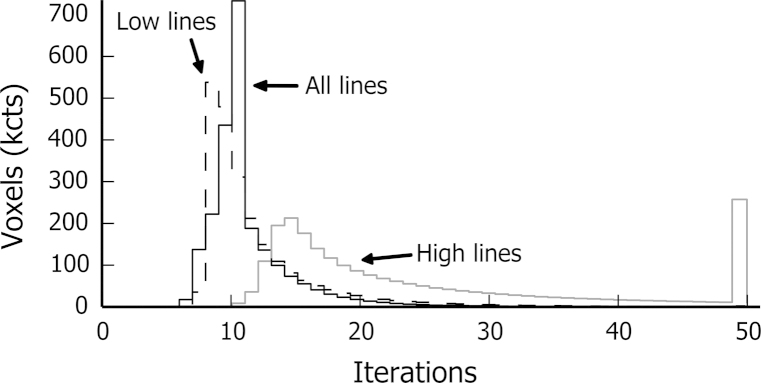
Iterations needed to solve the layer system for each voxel in the enhanced quantification. A histogram of the number of iterations needed to reach the convergence criterion to solve Eq. (1) is plotted for the whole set of data. The enhanced quantification is applied to the set of high, low or all energy lines.

To achieve further insight into the enhanced quantification, the layer system that is solved for each voxel in Eq. (3) is considered and the number of iterations needed to reach the convergence criterion is determined. The number of iterations is linked to the speed of convergence and gives an idea of the system complexity. The slower a system converges, the more likely it is to be ambiguous and the less likely the solution found is to be correct. Fig. 12 gives a histogram of the number of iterations needed for the enhanced quantification with the set of high, low or all energy lines. The layer system for the set of low energy lines converges with fewer iterations, as it is a system formed of fewer layers. Contrast that with the system of the set of high energy lines which, with more layers, converges after more iterations or does not converge up to the limit of iterations steps, set to 49. Using all lines helps to converge faster to a solution that is more likely to be correct, as confirmed by comparing maps c and d in Fig. 9.

**Fig. 13:**
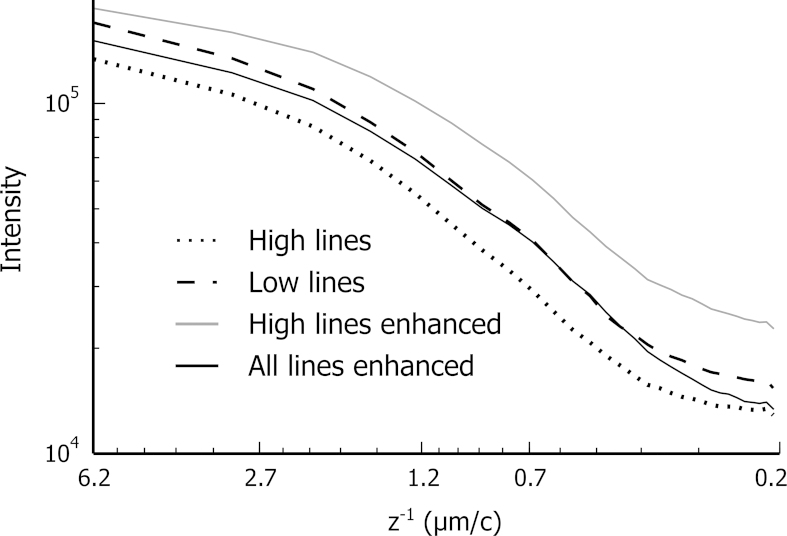
Measure of noise and edge sharpness of quantified Ni maps. A fast Fourier transform (FFT) is applied to the *z* axis of the 3D maps of Fig. 9. The *x* and *y* axes of the obtained power spectra are projected on to the spatial frequency (*z*^−1^) axis. The radius is given in μm per cycles and ranges from 6.2 to 0.2 μm, from the size of the stack to the distance between 2 voxels. The Ni content obtained with bulk and enhanced quantification are indicated with dashed/dotted and plain curves respectively. The dotted curve and the grey curve are quantified with the set of high lines, the dashed curve with the set of low lines, and the black plain curve with all energy lines.

The noise and the profile sharpness can be characterised in terms of spatial frequency (*z*^−1^) using a Fourier transform (FFT) applied to the *z* axis of a 3D map of Ni content obtained with the four quantifications of Fig. 9. Fig. 13 gives the projection of *x* and *y* axes on *z*^−1^ axis. The *z*^−1^ axis is given in microns per cycle, which corresponds to the inverse of the spatial frequency, and ranges from 6.2 to 0.2 μm, from the size of the stack to the distance between two voxels.

The range of influence of the enhanced quantification can be estimated by *r*_*max*_ of Eq. (3), the deepest voxel in which X-rays are generated, which is here 7 voxels (equivalent to 0.7 μm deep). In Fig. 13, around this value, the curve corresponding to the enhanced quantification with the high energy lines appears to be highest. This apparent increase in sharpness is in part due to the over-correction artefact, as can be observed in Fig. 13. The enhanced quantification with the set of all lines follows closely the curve for low energy lines (which should provide the best depth resolution). There is a gain in sharpness compared to the bulk quantification with high energy lines without the over-correction artefact.

The highest spatial frequencies, just below 0.2 μm, correspond to the noise voxel to voxel. In this region in Fig. 13, the four curves diverge, indicating different noise level. The highest noise level is obtained with the enhanced quantification with the high energy lines. The second highest is obtained quantifying with the low energy lines: the Ni Lα intensity is lower than Ni Kα (see Fig. 1) and the Ni Lα peak overlaps with the Co Lα peak. The noise level of the enhanced quantification with the set of all lines is close to the lowest noise level of the bulk quantification with high energy lines. Using the whole set of lines greatly helps to reduce the noise of the enhanced quantification.

## 5 Discussion

To discuss the enhanced quantification in more depth, the quality of the data needs to be considered first. In order to analyse a large volume in a reasonable total time, the time spent to record each spectrum must be limited to a few hundred milliseconds. Individual spectra necessarily have a relatively high noise level and provide only limited information. The dataset is however formed of millions of spectra measured on a few similar phases. This is a favourable case for statistical methods that can be used to reduce the noise to a required level for quantification.

In a first step, a running sum is used to reduce noise at the expense of spatial resolution. This is necessary to avoid generating an artefact (Hf, Ta overlap) with the second step, the principal component analysis (PCA). PCA generates a set of representative components that are fitted to the dataset as a linear sum. As shown in Fig. 8, each spectrum is significantly smoothed; the peaks are well defined and the background is less noisy. As *k*-ratios are background corrected values, their accuracy is greatly improved by a lower noise of both peak and background. The noise reduction obtained with PCA is complex to evaluate. It is proportional to the peak-to-background ratio of the peak as well as to the volume of the phase. Detecting a trace element homogeneously present in a main phase is then greatly improved. A minor element in a minor phase benefits less and may suffer from artefacts if the components are not carefully chosen, as observed with Hf and Ta. The noise reduction obtained with PCA benefits greatly from co-variant or partially co-variant signals such as families of X-ray lines, e.g. as Ni Kα and Ni Kβ, and X-ray lines from the same element, such as Ni Lα and Ni Kα. When the co-variance is strong between two X-ray lines, the signal in the PCA de-noised model is linked, the noise is lowered and the obtained intensity maps are partially mixed. Complete co-variance between high and low energy X-ray lines was observed in the case of TEM-based thin film EDS acquisition with low counts and negligible sample absorption [25]. In the present case, the partial co-variance between high and low energy X-ray lines is much weaker due to interaction volume effects and sufficiently high signal-to-noise ratio; this could be observed by comparing PCA applied on a the full EDS data set and PCA applied on a data set split between high and low energy. A more detailed discussion about PCA is outside the scope of this paper.

Experimental *k*-ratios are observed to be systematically higher than simulated values, especially for the low energy lines. This results in an analytical total (sum of all element compositions) greater than 100%, as observed in Fig. 11. This systematic behaviour can be explained by the difference in acquisition conditions between the standard and the unknown sample. The analysed sample surface is surrounded by trenches from whose surfaces spurious X-rays may be emitted that are added to the acquired spectrum. Due to the imprecision of the milling, the surface tilt of the sample may be different to that expected. Simulations approximately modelling the differences in acquisition have shown to reproduce qualitatively the global behaviour of the *k*-ratios. To improve the *k*-ratio accuracy, the acquisition of sample and standards should be modified, and standards should be recorded with the appropriate angle. Before the sample acquisition, a block should be extracted and placed on a support [26]. The obtained milled surface is then free from surrounding trenches and thus free of spurious X-rays. However, because block lift-out is a time-consuming method, the sample geometry described in this paper is often preferred and it is therefore important to show the robustness of the enhanced quantification applied to less than perfect data with possible trench-related artefacts.

To show that the enhanced quantification benefits from the higher spatial resolution arising from the set of low energy lines, and the improved quantification accuracy from the set of high energy lines, this method is compared to the classical quantification with either set of lines. As simulated in Fig. 1, 90% of the nickel X-rays are emitted from the first 50 nm, or from the first 300 nm, for Ni Lα and Ni Kα, respectively. In the depth direction, there is an important gain of spatial resolution for the low energy lines. This gain is almost totally retrieved using the enhanced quantification, as seen by the close match for lower frequencies in the Fourier transform figure (Fig. 13).

The most accurate composition is obtained using the classical quantification with high energy lines, as high energy lines are less sensitive to potential error from the absorption correction and to acquisition differences between sample and standard. The enhanced quantification gives a composition closer to the quantification with high energy lines than to the quantification with low energy lines, as seen in the difference in the mean analytical total of 13% and 21%, respectively (see Fig. 11). The enhanced quantification benefits from the accuracy of the high energy lines, but there is certainly room for improvement in reducing the 13% error to a value closer to zero.

The general drawback of any sharpening method is the likely increase in the noise level, as observed in Fig. 13 with the enhanced quantification with high energy lines. However, the noise level of the enhanced quantification with all lines is close to the lowest noise level of bulk quantification with high energy lines. The increase of noise is moderate and tolerable given the important gain in spatial resolution.

The accuracy of the enhanced quantification can be evaluated by considering one recursion step in two parts. The local structure of the sample is first approximated by a system of layers. The composition of the upper layer is the unknown of the system. In a second step, this system is solved with the XPP-*φ*(*ρz*) thin film quantification. This last point is improved in the present work. During the recursion, a system with layers sharing common elements is likely to be encountered. This is a known unfavourable case for thin film quantification [13]. The system is ambiguous and convergence to a local minimum is likely. In [14], the most unfavourable cases are avoided by grouping layers of similar composition together. The situation is improved, but inaccurate solutions still lead to an artefact of over-correction, as observed with simulated data (Fig. 7) and with experimental data (Fig. 9). The system can be made less ambiguous with additional *k*-ratios, either measured at different accelerating voltages or extracted from different energy lines. The second solution is successfully used in this work. With the full set of available lines, convergence is reached after significantly fewer iterations (see Fig. 12), indicating systems are globally less ambiguous. Consequently the over-correction artefacts are greatly reduced (see Fig. 9) and the result is faithful to the bulk quantification with low energy lines as observed with the Fourier transform in Fig. 13.

For further progress, data quality can be improved as well as the method itself. The angle of the milled surface can be carefully determined and used in the measurement of standard spectra. Alternatively, stage movement can be used to tilt the milled surface perpendicular to the electron beam. The enhanced quantification would then benefit from not needing a tilt correction. The data pre-processing could be further improved with improved low energy peak extraction. The top hat filter, used to remove the background, tends to reduce the energy resolution resulting in a less accurate peak deconvolution. Background fitting methods should prevent a worsening of the energy resolution, improving the later deconvolution. Absorption is not an isotropic phenomena since it takes place along the path towards the detector. This results in different X-ray intensity close to a boundary as observed with the contrast in the analytical-total maps in Fig. 11. The enhanced quantification could be improved by correcting absorption voxel by voxel instead of layer by layer as performed in the present work.

## 6 Conclusions

In a multi-element sample, if a sufficiently high accelerating voltage is used, two main X-rays lines of several elements are likely to be available for quantification. X-ray absorption in the sample for low energy lines results in an EDS map with higher spatial resolution but a lower quantification accuracy than for high energy lines. High energy lines are therefore usually preferred for quantification. This paper presents an enhanced quantification for 3D SEM–EDS data that uses all available X-ray energy lines, benefiting from the higher spatial resolution of the low energy set and the improved quantification accuracy of the high energy set. The method is recursive along the depth direction: at each step of the recursion, the influence of the deeper neighbouring voxels is corrected using the spatial information of the high and low energy lines.

The method is applied on data acquired from a Ni-based superalloy. The sample is rather complex with 11 elements, which were quantified using 15 X-ray lines, and with microstructural features revealed close to the 3D spatial resolution. The most common acquisition geometry is used, providing data with a relatively high level of noise and some acquisition artefacts. With the appropriate pre-processing, the enhanced quantification was shown to be as robust as the classical methods to noise and trench-related artefacts.

The method was demonstrated by comparison with other quantification methods. With the full set of X-ray lines, artefacts observed for the same method with the set of high energy lines are strongly reduced. The enhanced quantification benefits from the higher compositional accuracy of the high energy lines, although there is room for improvement. The noise increase is moderate, being below the noise level obtained with the low energy lines. This controlled increase of noise and the non-optimal quantification accuracy are balanced by the almost maximal gain in spatial resolution, close to that obtained with the low energy lines only.

## Appendix A Supplementary materials

Details of the 3D measurements. (a) The red surface, γ′ arms, is reconstructed from the stack of Ni content maps obtained with the enhanced quantification applied to all available lines. The green line indicates the position of a profile along z shown in (b). This profile goes through two arms of the γ′ phase (higher Ni content). The arms are extended in *x* and *y* direction and thin in the z direction. The different phase boundaries are approximately perpendicular to *z*. The SE contrast in dashed grey is adjusted between 0 and 1 (right scale). The Ni content obtained with bulk and enhanced quantification are indicated with dashed and plain curves respectively.
