# Supplementary figures and images for: Enhanced quantification for 3D SEM–EDS: Using the full set of available X-ray lines
Source: Ultramicroscopy. 2015 Jan;148:158–67. doi: 10.1016/j.ultramic.2014.10.010 (PMC4266451; doi:10.1016/j.ultramic.2014.10.010)

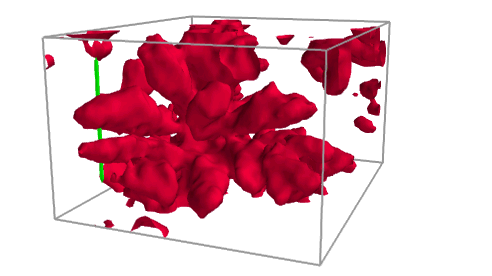

Supplement: Supplementary file 1 — Details of the 3D measurements. (a) The red surface, γ′ arms, is reconstructed from the stack of Ni content maps obtained with the enhanced quantification applied to all available lines. The green line indicates the position of a profile along z shown in (b). This profile goes through two arms of the γ′ phase (higher Ni content). The arms are extended in x and y direction and thin in the z direction. The different phase boundaries are approximately perpendicular to z. The SE contrast in dashed grey is adjusted between 0 and 1 (right scale). The Ni content obtained with bulk and enhanced quantification are indicated with dashed and plain curves respectively. [file mmc1.gif]
